# Effects of *BACE2* polymorphisms on verbal memory in the general population

**DOI:** 10.1007/s00406-026-02208-y

**Published:** 2026-03-28

**Authors:** Linda Garvert, Sarah Killer, Kevin Kirchner, Henry Völzke, Uwe Völker, Hans Jörgen Grabe, Sandra Van der Auwera

**Affiliations:** 1 Department of Psychiatry and Psychotherapy, University Medicine Greifswald, Greifswald, Germany; 2 DZHK (German Centre for Cardiovascular Research), Partner Site Greifswald, Greifswald, Germany; 3 Institute for Community Medicine, University Medicine Greifswald, Greifswald, Germany; 4 Interfaculty Institute for Genetics and Functional Genomics, University Medicine Greifswald, Greifswald, Germany; 5 German Centre for Neurodegenerative Diseases DZNE, Site Rostock/ Greifswald, Greifswald, Germany

**Keywords:** Amyloid β, Beta-site APP cleavage enzyme 2, SHIP-TREND study, APOE ε4, Cognitive decline, Preclinical Alzheimer's

## Abstract

Emerging evidence highlights the potential role of BACE2 (beta-site APP cleavage enzyme 2) in influencing Alzheimer’s disease (AD) risk and cognitive impairment, particularly due to its ability to cleave amyloid precursor protein (APP) at the θ-site in a non-amyloidogenic manner without the accumulation of neurotoxic Aβ_42_. Using data from the German population-based Study of Health in Pomerania (SHIP-TREND), we applied general linear regression models to evaluate the associations between 231 genetic variants within *BACE2* and two verbal memory scores in a cross-sectional (*N* = 3791) and a longitudinal setting (*N* = 2215). Additionally, we examined the moderating effect of *APOE* ε4 on the relationship between *BACE2* variants and memory performance. Three genetic clumps were significantly associated with verbal memory scores in the cross-sectional setting (p_FDR_<0.05). The minor T-allele of rs61736857 and the minor C-allele of rs1072869 were inversely associated with verbal memory, while the minor C-allele of rs6517661 showed a positive association. No interaction term with *APOE ε4* reached statistical significance in the cross-sectional setting. In longitudinal analyses, rs8133778 demonstrated a significant (p_FDR_<0.05) interaction effect with *APOE ε4* on memory. Genotype GG exhibited a more pronounced decline in verbal memory among ε4 carriers, while genotypes AA and AG represented a risk factor for non-carriers. We replicated some *BACE2* variants previously reported as potential risk factors in AD at a nominal significance level. In addition, our results identify novel variants that may contribute to cognitive impairment in the general population, some of which have been previously associated with altered BACE2 expression.

**Supplementary Information:**

The online version contains supplementary material available at 10.1007/s00406-026-02208-y.

## Introduction

Alzheimer’s disease (AD) is a progressive, multifactorial neurodegenerative disorder accounting for 60 to 80% of dementia cases worldwide [1, 2]. Major risk factors for AD include advanced age, genetic predisposition, and socioeconomic factors [3]. In addition to synaptic loss and cognitive decline [4], the pathological hallmarks of AD include the accumulation of extracellular amyloid-beta (Aβ) peptides and plaques [5].

The idea that Aβ plaque deposition is a primary driver of cognitive decline and AD pathogenesis was first proposed in the 1980s and subsequently led to the amyloid cascade hypothesis by Hardy and Higgins [6]. Aβ is generated from the amyloid precursor protein (APP) through enzymatic cleavage by secretases [7], resulting in protease-resistant peptide aggregates accumulating inside and outside of neurons [8].

Research on Aβ aggregation has focused on genetic mutations in *APP* [9] or genes encoding APP-processing enzymes that may influence Aβ levels in the brain [10]. One such enzyme is the beta-site APP cleavage enzyme 1 (BACE1; [11, 12]), which produces the amyloidogenic and neurotoxic Aβ_42_ monomer [13]. Another protease of interest is the beta-site APP cleavage enzyme 2 (BACE2), which has gained increased interest due to its homology with BACE1 and its potential involvement in cognition and AD. Similar to BACE1, BACE2 is capable of generating the neurotoxic Aβ_42_ peptide. However, it also facilitates the production of non-amyloidogenic Aβ variants such as Aβ_19_, Aβ_20,_ and Aβ_34_ by cleaving at an alternative APP site, leading to lower levels of the neurotoxic Aβ_42_ monomer [1]. Notably, BACE2 is located on chromosome 21, like APP. The observation that only 70% of individuals with Down syndrome (DS, Trisomy 21) develop early signs of AD despite harbouring three copies of *APP* suggests the presence of a neuroprotective genetic factor located on chromosome 21 that counteracts the neurotoxic APP processing. In recent years, BACE2 has emerged as a promising neuroprotective candidate in the context of AD and cognitive impairment [1, 10].

Genetic studies on *BACE2* have primarily focused on selected candidate variants and specific disease populations, such as individuals with AD or DS. Myllykangas and colleagues [14] identified an association between two *BACE2* three-site haplotypes and AD in two independent cohorts (population-based and AD vs. controls). Furthermore, they identified a single nucleotide polymorphism (SNP, rs2252576) associated with neuropathologically verified AD in the population-based cohort. This SNP, among other associations, was later replicated in an independent DS cohort of 67 subjects by Mok and colleagues [15], who investigated multiple SNPs in relation to age at onset of dementia. In a larger cohort, Huentelman and colleagues [10] analysed SNPs across the entire *BACE2* gene in an AD case-control sample from the Alzheimer’s Disease Neuroimaging Initiative (ADNI) cohort. They identified several SNPs associated with late-onset AD, particularly in apolipoprotein E ε4 (*APOE* ε4) non-carriers. In large genome-wide association studies (GWAS), *BACE2* was significantly associated with cognition-related traits such as memory and educational attainment [16–18]. Two GWAS on educational attainment identified genome-wide significant variants within *BACE2*. Extended analyses also identified associations with related traits such as mathematical ability and cognitive performance, the latter encompassing results from multiple cognitive ability tests [16]. In a smaller South African cohort of 2,246 individuals, BACE2 showed a significant gene-based association with overall cognition, spanning several domains including language ability, executive function, and verbal memory [18]. However, other large-scale genetic studies on verbal memory have reported no evidence supporting an involvement of BACE2 [19, 20].

However, it remains unclear to what extent genetic variation within *BACE2* influences the development of cognitive impairment in the general population without a clinical AD diagnosis. Such an effect is conceivable, considering the role of BACE2 in Aβ production and its previously reported associations in specific disease populations.

The aim of the current study is therefore to analyze the impact of *BACE2* genetic variants on verbal memory performance in the general adult population using data from the SHIP-TREND study [21] in both cross-sectional and longitudinal analyses. Variants across the entire *BACE2* gene will be examined to replicate previous findings and identify novel candidate variants. To account for previously reported differences in *APOE* ε4 carriers and non-carriers [10], moderation analyses will be conducted. Our results may contribute to a better understanding of the relevance of BACE2 in future research on cognitive decline and early-stage AD.

## Materials and methods

### Study population

Data from the TREND cohort of the Study of Health in Pomerania (SHIP) were used [21]. The target population comprised adult German residents of northeastern Germany who lived in three cities and 29 communities, with a total population of 212,157. Employing a two-stage stratified cluster sampling method, adults aged 20 to 79 years (baseline) were randomly drawn from local population registration files in which every resident has to be included by law. From the initial net sample of 8016 eligible subjects, 4420 Caucasian individuals participated at baseline (SHIP-TREND-0; hereafter referred to as TREND-0) between 2008 and 2012. From 2016 to 2019, a follow-up study, called SHIP-TREND-1 (TREND-1), was conducted, which included similar assessments to those in TREND-0. This study involved 2507 subjects from the baseline TREND-0 sample. All participants in TREND-0 and TREND-1 underwent a standardized computer-assisted personal interview, during which they conducted verbal memory tests and provided information on sociodemographic and lifestyle factors.

The investigations in SHIP-TREND were carried out in accordance with the Declaration of Helsinki, including written informed consent of all participants. Survey and study methods were approved by the institutional review boards of the University Medicine Greifswald (Germany).

### Phenotype measures

#### Verbal memory

The Nuremberg Age Inventory (NAI) word list was used to measure immediate and delayed verbal memory performance. The NAI is a German test developed to assess cognitive abilities during brain aging [22, 23]. To measure verbal memory performance, participants are read a list of eight words, which they are asked to repeat immediately afterwards. The score for immediate word recall (NAI_recall_) is the total number of correctly repeated words. After 20 min, participants are asked to identify the previously heard words from another word list, including the original 8 words and 8 additional distractor words. The score for delayed word recognition (NAI_recognition_) is obtained by subtracting the number of wrongly identified distractor words from the number of correctly recognized words. For the longitudinal NAI score trajectories, NAI scores from the baseline assessment were subtracted from the respective scores in the follow-up. Therefore, negative NAI_recall_ or NAI_recognition_ trajectories represent a decline in verbal memory performance.

#### Covariates

On the phenotypic level, we adjusted for age, sex, current depressive symptoms, and education. Depressive symptoms in the two weeks before the assessments were measured using the Patient Health Questionnaire (PHQ-9). The PHQ-9 is a 9-item self-report questionnaire with high reliability and validity [24], with higher values indicating a higher load of current depressive symptoms. Education was measured by years of schooling and categorized into three levels according to the German school system: less than ten years, exactly ten years, and more than ten years of schooling.

### Genomic data

#### Genotyping and imputation

Information on single nucleotide polymorphisms was derived from the genetic data in TREND. Genotyping of a subset of the TREND subjects (*N* = 986) was conducted using the Illumina Infinium HumanOmni 2.5 Bead Chip. The remaining TREND sample (*N* = 3133) was genotyped using the Illumina Infinium GSA. SNPs with a Hardy-Weinberg equilibrium (HWE) *p*-value < 0.0001, a call rate < 0.95, as well as monomorphic SNPs and singletons, were excluded prior to imputation. Due to the different genotyping platforms, we employed a two-step imputation workflow that minimizes array-specific batch effects [25]. In this method, the datasets are first imputed pairwise against each other, and a panel of overlapping imputed variants is created. This intermediate panel was then imputed against the 1000 Genomes Phase 3 (Version 5) reference panel using the Michigan imputation server with Eagle2 for phasing [26, 27].

#### SNP selection

We selected all single nucleotide polymorphisms (SNPs) within the *BACE2* gene (chr21: 42540087–42654457, build37), including 5 kb upstream and downstream, with a minor allele frequency (MAF) of > 5%, imputation quality > 0.8, and a genotype missingness of less than 5%. This resulted in 231 SNPs available in TREND for analysis.

#### APOE ε4 carrier status

The *APOE* ε4 carrier status was inferred from the genotypes of two SNPs, rs429358 (C; T) and rs7412 (T; C) [28, 29]. Since this information was obtained from genome-wide SNP array data instead of strand-specific targeted genotyping, certain haplotype combinations - specifically *APOE* ε1/ε3 and ε2/ε4 - could not be differentiated [30]. Participants with these genotype combinations were excluded from the analyses (*N* = 99). Subjects with at least one ε4-allele were classified as *APOE* ε4 carriers.

### Statistical analyses

Sample characteristics of the final study sample were assessed by median, interquartile range, and minimum-maximum range for metric variables and by counts and percentages for categorical data. Significant differences between *APOE* ε4 carriers and non-carriers were tested via Wilcoxon rank sum test for metric variables and Pearson’s Chi-squared test for categorical data.

Generalized linear regression models were employed to investigate the association between genetic variation within *BACE2* and the two verbal memory scores, NAI_recall_ and NAI_recognition_, as well as their respective trajectories. In addition to these four models assessing direct SNP effects, we conducted four equivalent models incorporating the interaction term SNP × *APOE* ε4 carrier status as the primary predictor.

The cross-sectional models for NAI_recall_ were modeled using a binomial distribution. The remaining outcome variables were shifted to ensure non-negativity and modeled with a quasi-Poisson distribution.

All regression models were adjusted for previously selected confounding factors. Specifically, the cross-sectional models (both direct and interaction) for NAI_recall_ and NAI_recognition_ were adjusted for sex, age, age², *APOE* ε4 carrier status, age² × *APOE* ε4 carrier status, education, PHQ-9, and the first five genetic principal components.

For the longitudinal models (both direct and interaction) assessing the trajectories of NAI_recall_ and NAI_recognition_, we adjusted for the same variables, with the addition of PHQ-9 at follow-up, the respective baseline NAI score, and the time (in years) between assessments. Age was centered prior to analysis.

To ensure sufficient statistical power, especially in interaction analyses, it is essential to maintain adequate sample sizes within each genotype group. Therefore, a dominant genetic model was applied for SNPs with fewer than 400 individuals homozygous for the minor allele, combining homozygous minor allele carriers and heterozygous allele carriers into a single group. For all other SNPs, an additive model was employed.

For each of the eight models, results were clumped in PLINK 1.9 [31] with a 100 kb window and linkage disequilibrium (LD) threshold r² = 0.1. We selected the lead SNP for each clump and applied false discovery rate (FDR) correction to their p-values to correct for the number of independent tests. All reported analyses were performed with R version 4.4.3 [32].

### Functional annotation of significant SNPs

For each of the SNPs showing a nominal significant association in at least one of the eight models, we derived several indices suggesting functionality using Functional Mapping and Annotation of Genome-Wide Association Studies (FUMA [33]); (a) annotations with ANNOVAR [34]; (b) Combined Annotation Dependent Depletion (CADD) score (http://cadd.gs.washington.edu/*)* that reflects deleteriousness of variants computed by integrating 63 functional annotations. A higher CADD score suggests a more deleterious SNP. The previously suggested threshold for deleteriousness is 12.37 [35, 36]; (c) RegulomeDB scores indicating the level of evidence for a variant to be a regulatory element. It is a categorical score from 1a to 7, with 1a being the highest score that suggests the most biological evidence to be a regulatory element [37]; (d) the minimum 15-core chromatin states across 127 tissues/cell types [38, 39]; (e) the most common 15-core chromatin states across 127 tissues/cell types.

In addition, we used the Genotype-Tissue Expression (GTEx) Portal (v10) (https://www.gtexportal.org/home) [40] to identify which of the nominally significant SNPs had previously been reported as expression quantitative trait locus (eQTL) in any of the 13 tested brain tissues ( amygdala, anterior cingulate cortex, caudate (basal ganglia), cerebellar hemisphere, cerebellum, cortex, frontal cortex, hippocampus, hypothalamus, nucleus accumbens (basal ganglia), putamen (basal ganglia), spinal cord (cervical c-1), and substantia nigra) . Lastly, we queried all nominally significant variants in the NHGRI-EBI GWAS Catalog [41, 52] and the IEU OpenGWAS project [42] to identify previously reported genome-wide significant (*p* < 5 × 10^− 8^) associations.

## Results

A sample description of TREND-0 participants is presented in Table 1, and detailed information on the analyzed SNPs is provided in Supplementary Table S1. The final sample comprised 3791 subjects with a median age of 52 years, 52% of whom were female, and 24% were *APOE* ε4 carriers. No statistical differences were observed between carriers and non-carriers for any of the analyzed outcomes and covariates (Table 1). A description of the follow-up TREND-1 sample (*N* = 2215) is shown in Supplementary Table S2. Again, no differences were detected between *APOE* ε4 carriers and non-carriers. However, the subset of participants attending the follow-up assessment was younger at baseline and had higher NAI_recall_ scores compared to the complete baseline TREND-0 sample.

**Table 1:** Sample characteristics of the 3791 TREND-0 participants and the subgroups of *APOE* ε4 carriers and non-carriers

Characteristic	Complete sample	APOE ε4 carrier status		
	*N* = 3,791^1^	Non-carrier *N* = 2,881^1^	Carrier *N* = 910^1^	*p*-value^2^
Age (in years)	52 (40, 64) [20–83]	52 (40, 64) [20–83]	51 (39, 63) [20–82]	0.075
Sex				> 0.9
Male	1,831 (48%)	1,390 (48%)	441 (48%)	
Female	1,960 (52%)	1,491 (52%)	469 (52%)	
NAI recall	5 (4, 6) [0–8]	5 (4, 6) [0–8]	5 (4, 6) [0–8]	> 0.9
NAI recognition	6 (5, 7) [-3-8]	6 (5, 7) [-3-8]	6 (5, 7) [-1-8]	0.8
Unknown	23	17	6	
Education				0.083
< 10 years	832 (22%)	646 (22%)	186 (20%)	
10 years	1,986 (52%)	1,480 (51%)	506 (56%)	
> 10 years	973 (26%)	755 (26%)	218 (24%)	
PHQ-9	3 (1, 6) [0–26]	3 (1, 6) [0–26]	3 (1, 6) [0–26]	0.5
APOE ε4				
Non-carrier	2,881 (76%)			
Carrier	910 (24%)			

For metric variables, median, interquartile range, and minimum-maximum are reported, for categorical variables, counts and percentages

^1^continuous: Median (Interquartile range Q1,Q3) [Minimum - Maximum]; categorical: counts (percentages)

^2^continuous: Wilcoxon rank sum test; categorical: Pearson’s Chi-squared test

NAI: Nuremberg Age Inventory; PHQ-9: Patient Health Questionnaire

### Cross-sectional analyses in TREND-0

#### Direct SNP effects

In direct association analyses between 231 *BACE2* SNPs and NAI_recall_ in TREND-0 (*N* = 3791), we identified 19 independent clumps (clusters of SNPs in LD) with a total of 13 nominally significant SNPs. Of the 19 lead SNPs, 3 were nominally significant (rs111773851, rs2012050, rs12483323) and one was significant after FDR correction for the number of independent clumps (p_FDR_ < 0.05). The minor T allele of rs61736857 was significantly associated with a lower NAI_recall_ score (beta = -0.08, 95%CI = [-0.132, -0.029], *p* = 0.0022).

**Fig. 1:**
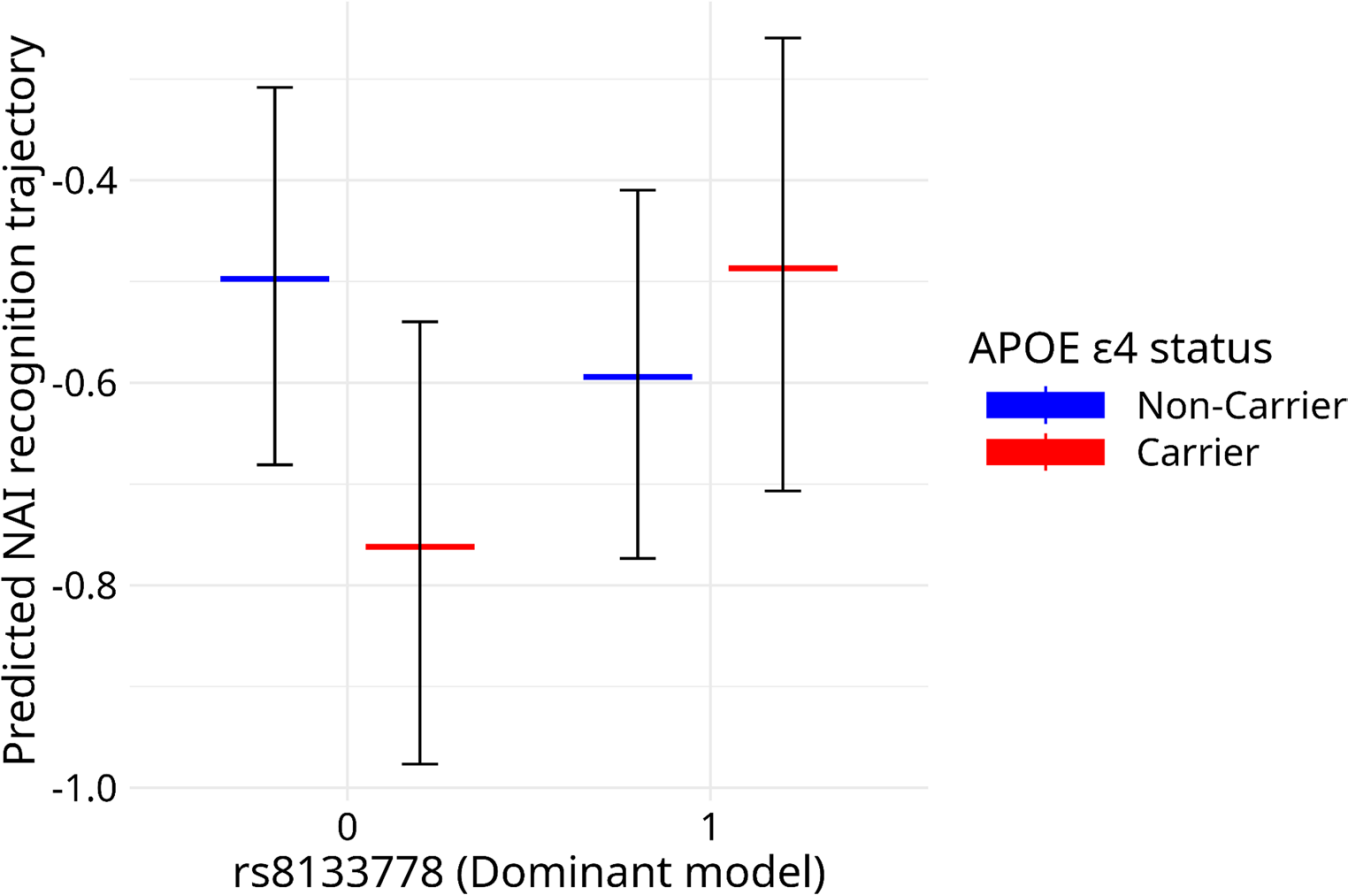
Interaction effect between rs8133778 and *APOE ε4* carrier status on the predicted NAI recognition trajectory, when co-variables are set to their mean (numeric) or the reference level (factor). The plot shows the predicted value and 95% confidence interval. Dominant genetic model with GG (0) vs. AG/AA (1)

For NAI_recognition_, 17 clumps were identified with a total of 23 nominally significant SNPs. Two lead SNPs, rs1072869 and rs6517661, were significant after FDR correction for 17 clumps. The minor C allele of rs1072869 was significantly associated with lower verbal memory scores (beta = -0.015, 95%CI = [-0.024, -0.007], *p* = 0.0005) and the minor C allele of rs6517661 with higher scores (beta = 0.021, 95%CI = [0.006, 0.035], *p* = 0.0043). In addition, lead SNP rs111773851 reached nominal significance. Results for all nominally significant lead SNPs are shown in Table 2; Fig. 1; results for all variants are presented in Supplementary Table S3.

#### Interaction with *APOE* ε4 carrier status

To account for previously observed differences between *APOE* ε4 carriers and non-carriers, we examined the moderating effects of *APOE *ε4 on the association between *BACE2* SNPs and verbal memory. In our cross-sectional analysis, none of the 231 *BACE2* SNPs tested demonstrated a statistically significant interaction with *APOE* ε4 carrier status in relation to verbal memory. From the 17 clumps identified for NAI_recall_ and the 16 clumps for NAI_recognition,_ three and two lead SNPs, respectively, reached nominal significance, with a total of five SNPs for NAI_recall_ and eleven for NAI_recognition_ showing nominal significance. Results for nominally significant lead SNPs are shown in Table 2; Fig. 1; results for all variants are presented in Supplementary Table S3.

**Table 2:** Nominally significant lead SNPs in direct and interaction analyses on verbal memory in TREND-0 (N_recall_ = 3791, N_recognition_ = 3768)

Outcome	Clump	rsID	Alleles^1^	Dom^2^	Effect^3^	*P* ^4^
**Direct effects**						
NAI recall	1	rs61736857	T/C	T	-0.08 [-0.132, -0.029]	**0.0022**
NAI recall	2	rs111773851	T/C	T	-0.096 [-0.165, -0.026]	0.0072
NAI recall	3	rs2012050	T/C	F	0.04 [0.005, 0.076]	0.0269
NAI recall	4	rs12483323	T/C	T	-0.059 [-0.112, -0.005]	0.0315
NAI recognition	1	rs1072869	C/A	F	-0.015 [-0.024, -0.007]	**0.0005**
NAI recognition	2	rs6517661	C/A	T	0.021 [0.006, 0.035]	**0.0043**
NAI recognition	3	rs111773851	T/C	T	-0.018 [-0.035, -0.001]	0.0425
**Interaction effects (SNP × APOE ε4)**						
NAI recall	1	rs2837997	T/C	T	-0.176 [-0.332, -0.019]	0.0279
NAI recall	2	rs74742373	A/G	T	0.14 [0.011, 0.27]	0.0340
NAI recall	3	rs111773851	T/C	T	-0.166 [-0.327, -0.004]	0.0439
NAI recognition	1	rs9983699	C/T	T	0.035 [0.007, 0.064]	0.0156
NAI recognition	2	rs914185	T/C	T	0.032 [0.002, 0.061]	0.0381

^1^Effect allele / non-effect allele

^2^Use of dominant model: T(RUE), F(ALSE)

^3^Estimate [95% confidence interval]

^4^Uncorrected association p-value; bold: significant after FDR-correction

### Longitudinal analyses in TREND-0 and TREND-1

#### Direct SNP effects

The longitudinal analyses of memory trajectories between TREND-0 and TREND-1 were conducted on a smaller sample (*N* = 2215) due to incomplete participation at follow-up. The median follow-up time between the TREND-0 and TREND-1 assessments was 7.3 years. No FDR-corrected significant effects were observed for the direct effects of *BACE2* SNPs on verbal memory trajectories. Analyses of the NAI_recall_ trajectory yielded 15 independent clumps with three nominally significant lead SNPs (rs12482462, rs35470608, rs112097565) among a total of 14 nominally significant SNPs. For the NAI_recognition_ trajectory, three out of 14 clumps displayed nominally significant lead SNPs (rs116538541, rs28654619, rs914186). In total, 7 SNPs reached nominal significance. Results for all nominally significant lead SNPs are shown in Table 3; Fig. 1; results for all variants are presented in Supplementary Table S4.

#### Interaction with *APOE* ε4 carrier status

In the interaction analysis examining whether *APOE* ε4 moderates the association between *BACE2* SNPs and the NAI_recall_ trajectory, one lead SNP (rs8127120) out of 18 clumps reached nominal significance, with a total of three SNPs showing nominal significant effects. Analysis of the NAI_recognition_ trajectory revealed 27 nominally significant interactions, with two nominally significant lead SNPs (rs2009135 and rs2837966). Another lead SNP (rs8133778) remained significant after correction for 17 independent clumps. Lead SNP rs8133778 exhibited a statistically significant interaction effect with *APOE* ε4 on the NAI_recognition_ trajectory (beta = 0.058, 95%CI = [0.02, 0.096], *p* = 0.0027). In *APOE* ε4 carriers, individuals with the GG genotype of rs8133778 showed a more substantial decline in verbal memory compared to those with AA or AG genotypes. Conversely, in *APOE* ε4 non-carriers, genotypes AA/AG were associated with a more pronounced decline in NAI_recognition_ (Fig. 1). Results for all nominally significant lead SNPs are shown in Table 3; Fig. 1; results for all variants are presented in Supplementary Table S4.

**Table 3:** Nominally significant lead SNPs in direct and interaction analyses on verbal memory trajectories between TREND-0 and TREND-1 (N_recall_ = 2215, N_recognition_ = 2195)

Outcome	Clump	rsID	Alleles^1^	Dom^2^	Effect^3^	*P* ^4^
**Direct effects**						
NAI recall	1	rs12482462	T/C	F	-0.015 [-0.027, -0.003]	0.0164
NAI recall	2	rs35470608	C/T	T	0.021 [0.003, 0.039]	0.0220
NAI recall	3	rs112097565	A/G	T	-0.02 [-0.037, -0.003]	0.0231
NAI recognition	1	rs116538541	C/A	T	-0.023 [-0.041, -0.004]	0.0150
NAI recognition	2	rs28654619	C/T	T	0.02 [0.003, 0.037]	0.0181
NAI recognition	3	rs914186	G/A	F	0.014 [0.002, 0.025]	0.0268
**Interaction effects (SNP × APOE ε4)**						
NAI recall	1	rs8127120	G/A	T	0.052 [0.005, 0.1]	0.0297
NAI recognition	1	rs8133778	A/G	T	0.058 [0.02, 0.096]	**0.0027**
NAI recognition	2	rs2009135	G/A	T	-0.052 [-0.09, -0.014]	0.0069
NAI recognition	3	rs2837966	G/C	T	0.052 [0.009, 0.094]	0.0166

^1^Effect allele / non-effect allele

^2^Use of dominant model: T(RUE), F(ALSE)

^3^Estimate [95% confidence interval]

^4^Uncorrected association p-value; bold: significant after FDR-correction

### Comparison of results

**Fig. 2:**
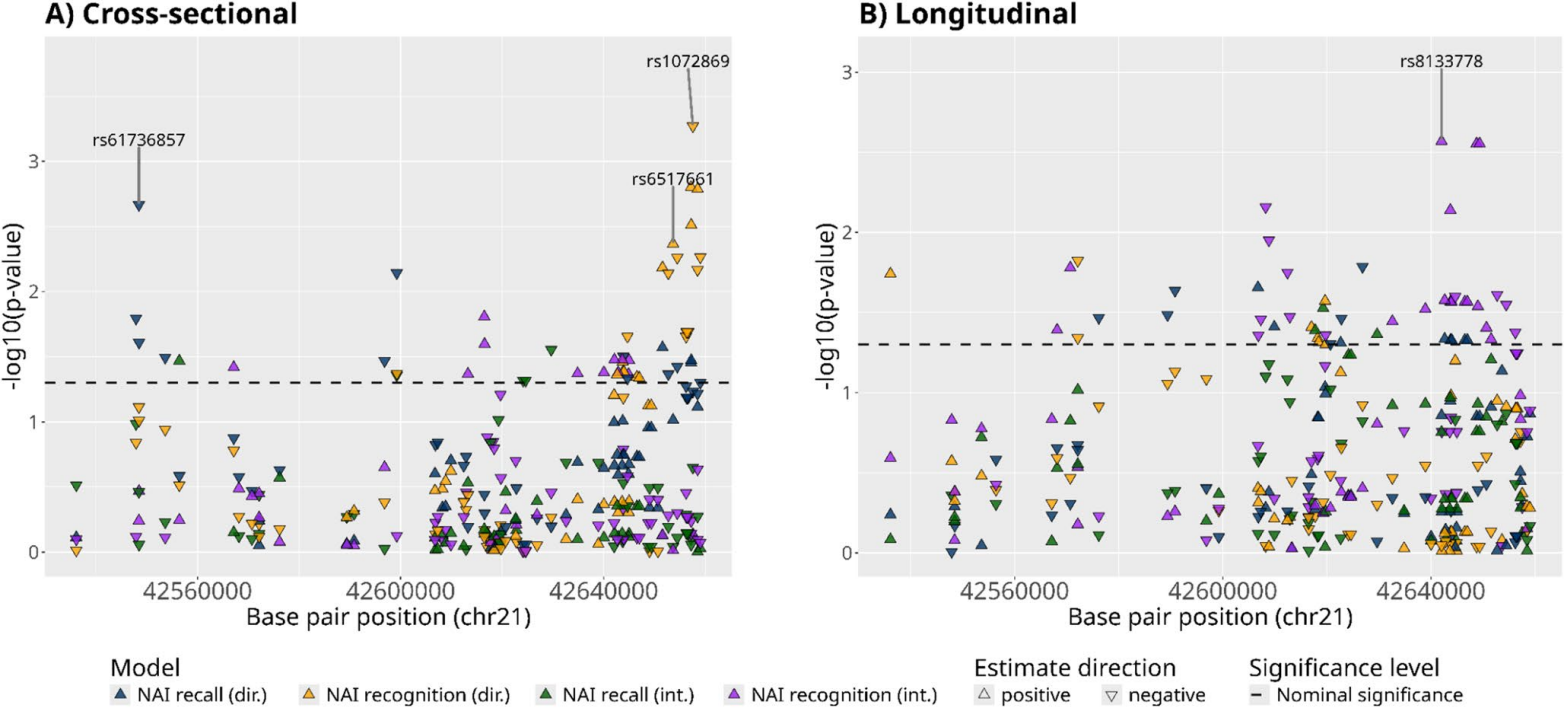
Manhattan plot for *BACE2* SNP associations with verbal memory in the different analysis models. The four colors indicate the model outcome (NAI_recall_, NAI_recognition_) and whether direct or interaction effects are displayed. The direction of effect is shown by upwards (positive) and downwards (negative) pointing triangles. The plot displays only variants with nominal significance in at least one of the eight models. FDR-corrected significant lead SNPs are named by rsID (compare Tables 2 and 3). The dashed line indicates nominal significance (*p* < 0.05). **A** Results for the cross-sectional analyses in TREND-0 (*N* = 3791). **B** Results for the longitudinal memory trajectories (*N* = 2215)

To compare results across analyses, we compiled all SNPs that reached nominal significance in any of the eight models (4 cross-sectional and 4 longitudinal models). Manhattan plots (Fig. 2) show more nominally significant SNPs located at the end of the *BACE2* gene, especially for analyses focusing on NAI_recognition_. Among the 78 *BACE2* SNPs that were nominally significant in at least one analysis, 10 variants were nominally significant in two or three analyses (Supplementary Figure S1). For example, the minor allele of rs111773851 was negatively associated with both NAI_recall_ and NAI_recognition_ in the cross-sectional setting and further exhibited a negative interaction with *APOE* ε4 on NAI_recall_ in the cross-sectional analysis. Similarly, the minor alleles of rs58867243, rs8126886 and rs79171038 were negatively associated with NAI_recall_ and NAI_recognition_ in the cross-sectional models and showed a negative interaction with *APOE* ε4 in the longitudinal analysis of NAI_recognition_. In contrast, the minor allele of rs2012050 demonstrated significant positive associations across these three models. Overall, the effect directions for the minor alleles of the 78 nominally significant SNPs were heterogeneous, with both positive and negative associations observed across models.

### Functional annotation of significant SNPs

The functionality indices for all 78 nominally significant variants are summarized in Supplementary Table S5. The CADD score ranges from 0.01 to 10.4, hence no variant surpassed the CADD score threshold of 12.37 for deleteriousness. The RegulomeDB score ranges from 3a (suggestive evidence for regulatory function) to 7 (no known evidence for regulatory function).

Based on data from the GTEx Portal v10, 23 of the 78 SNPs are known eQTLs in brain tissues (Supplementary Table S6). They affect the expression of *PLAC4* in the spinal cord (cervical c-1), *C2CD2* in the cortex, *LINC00232* in all of the 13 tested brain tissues except for the cortex, and *BACE2* in all 13 tested brain tissues.

Of the 78 nominally significant variants, 62 show genome-wide significant associations with 26 unique traits reported in either the GWAS Catalog or the IEU OpenGWAS project (Supplementary Table S7). These traits include, for example, cognitive performance (MTAG), educational attainment, years of schooling, diffusion MRI measures, as well as circulating protein levels such as FAM3B and BACE2.

## Discussion

In this study, we analyzed data from 3791 participants in the population-based TREND-0 baseline study and 2215 participants who also participated in the follow-up TREND-1 study. Within both waves, we investigated the effects of 231 genetic variants in the *BACE2* gene and their interaction with *APOE* ε4 carrier status on verbal memory using a cross-sectional design in TREND-0, as well as longitudinal analyses of memory trajectories between TREND-0 and TREND-1. We identified specific genetic variants associated with verbal memory scores, which may serve as early indicators of mild cognitive impairment and preclinical AD.

In recent years, *BACE2* has been proposed as a candidate gene for a neuroprotective amyloid pathway in AD [1, 43] due to its alternative APP cleaving activity. Several studies have demonstrated the protective effects of BACE2, highlighting its ability to cleave the amyloid precursor protein in a non-amyloidogenic way, thereby reducing the formation of neurotoxic Aβ_42_ peptides. Overexpression studies in various cell lines have shown that BACE2 contributes to the degradation of toxic Aβ peptides [44–46]. However, studies in individuals with DS have failed to establish a general protective effect of BACE2 overexpression, suggesting that individual genetic variability at the SNP level within *BACE2* may play a role [1].

Previous research in human populations has shown an impact of *BACE2* genetic variants on AD susceptibility and age at onset [10, 14, 15]. Mok and colleagues examined 83 *BACE2* SNPs in a sample of Down Syndrome individuals diagnosed with AD to determine their genetic contribution to AD age of onset [15]. Although six SNPs reached nominal significance in their study, none of these reached nominal significance in our direct SNP association models, with two of these SNPs not available in our data due to low MAF. However, rs2837990 narrowly missed significance in our *APOE* ε4 interaction model for the NAI_recall_ trajectory (*p* = 0.05). Differences in sample composition, analytic approach, or statistical modeling may account for these differences. Similarly, a study by Myllykangas et al. classified nearly 150 individuals from an ageing cohort in Finland into AD cases and controls [14]. Using a haplotype approach that included four SNPs, they tested for an association with AD. Although we used a single SNP approach, we identified one of these SNPs, rs1467756, reaching nominal significance in our direct association model for the NAI_recall_ trajectory. Additionally, Huentelman and colleagues conducted a comprehensive analysis across the entire *BACE2* gene, also investigating SNP interaction with *APOE* ε4 carrier status and their effects on AD [10]. Several SNPs identified in their study also demonstrated nominally significant associations in our analyses. Notably, the SNP ***×*** *APOE* ε4 interactions for rs9980146 and rs2012050, previously linked to cerebrospinal fluid levels of Aβ_42_ in individuals with mild cognitive impairment, were significantly associated with both verbal memory scores in our SHIP-TREND-0 baseline sample. Furthermore, two SNPs associated with AD status in the study by Huentelman and colleagues, rs2837994 and rs8134992, were successfully replicated in our *APOE* ε4 interaction models on the nominal significance level.

These prior studies were based on relatively small sample sizes and presented highly selected samples and analytic frameworks regarding SNP selection, moderating factors, and subanalyses. However, *BACE2* SNPs have also been linked to neurodegenerative and cognitive performance traits at a genome-wide level, including educational attainment, cognitive performance, math ability [16, 47], and AD [48, 49], as well as to obesity-related traits [50, 51]. To place our results in context, we cross-checked the nominally significant SNPs identified in our study against reported associations in the NHGRI-EBI GWAS Catalog and the IEU OpenGWAS project [41, 52]. Variants rs9981747, rs960231, rs914187, rs914186, and rs12482462 are listed as genome-wide significant for cognitive and mathematical ability as well as educational attainment [16, 17] (Supplementary Tables S7). Moreover, variant rs28654619, associated with the NAI_recognition_ trajectory, is genome-wide significant for several phenotypes related to diffusion MRI [53], indicating strong associations with intrinsic connectivity and white matter integrity in key brain regions, including the cerebellar peduncle and internal capsule areas. Lastly, variant rs61735054, associated with NAI_recall_, has been previously shown to be genome-wide significant for the thickness of the left hemisphere precuneus [54]. Other recent GWAS on AD or small vessel disease [55–57] did not report a significant contribution of *BACE2* variants.

The SNPs identified in our study are not only pre-described due to their association with cognitive traits; but several of them also function as eQTL in various tissues, with 23 specifically expressed in brain tissues (Supplementary Table S6). According to data from the GTEx portal, nominal significant variants from our analyses affect BACE2 levels in each of the 13 tested brain tissues. However, not all of the alleles associated with increased BACE2 levels are associated with positive effects on verbal memory in our study. For example, while variants such as rs2837984, rs2252943, rs9981553 and rs960231 suggest a relation between higher BACE2 levels and improved memory in the longitudinal analyses, other variants such as rs12483323, rs58867243, and rs58854288 indicate a relation between higher BACE2 levels in the brain and reduced memory scores in the cross-sectional analyses. Therefore, our results do not consistently support the hypothesis of a neuroprotective function of BACE2.

The observed interactions between *BACE2* variants and *APOE* ε4 carrier status also raise questions about potential molecular mechanisms involving both factors. Previous analyses have shown that the BACE2 enzyme can cleave APP in both amyloidogenic and non-amyloidogenic pathways, and several mechanisms are known to influence BACE2 activity. For example, mutations in the membrane domain of APP can alter its three-dimensional structure, thereby promoting its amyloidogenic function. Additionally, the binding of clusterin - a neuronal chaperone protein whose levels increase in older age and are elevated in AD and DS patients - at that domain further enhances the amyloidogenic activity of BACE2 [58]. The *APOE* ε4 genotype can impact the clusterin concentration in the synapse. Higher levels of clusterin may lead to increased binding to APP, which in turn enhances the amyloidogenic processing by BACE2.

While our results partially support *BACE2* as a potential candidate gene for verbal memory decline in the general population, our study provides several methodological advantages over previous research. The SHIP-TREND cohort comprises a large population-based sample with standardized cognitive assessments and regular follow-ups, facilitating the investigation of longitudinal effects. Verbal memory scores served as indicators of early cognitive impairment, and the genetic analyses encompassed SNPs throughout the entire *BACE2* gene, including interactions with *APOE* ε4 carrier status, which denotes subjects with an increased genetic risk for AD. However, a limitation of our study is its association-based design, which lacks direct biological insights into BACE2 function, and the absence of a replication sample, making it challenging to generalize the findings.

Overall, our analyses identified associations between *BACE2* genetic variants and verbal memory in different temporal and *APOE* ε4 interaction models. We identified four lead SNPs, rs61736857, rs1072869, rs6517661, and rs8133778, with statistically significant effects on verbal memory, as well as several nominally significant variants, some of which were previously reported for AD or cognition-related traits. Our findings do not provide clear evidence regarding potential neuroprotective effects of BACE2, and further studies are required to clarify its role. Since BACE2 remains a plausible candidate in AD pathogenesis, further research is necessary to identify possible interactions with other factors, such as clusterin or APP, and to clarify its overall role in neurodegeneration and its specific contribution to AD etiology.

## Supplementary Information

Below is the link to the electronic supplementary material.

Supplementary Material 1
